# Phase-related differences in egg production of the migratory locust regulated by differential oosorption through microRNA-34 targeting *activinβ*

**DOI:** 10.1371/journal.pgen.1009174

**Published:** 2021-01-06

**Authors:** Lianfeng Zhao, Wei Guo, Feng Jiang, Jing He, Hongran Liu, Juan Song, Dan Yu, Le Kang

**Affiliations:** 1 Beijing Institutes of Life Science, Chinese Academy of Sciences, Beijing, China; 2 CAS Center for Excellence in Biotic Interactions, University of Chinese Academy of Sciences, Beijing, China; 3 State Key Laboratory of Integrated Management of Pest Insects and Rodents, Institute of Zoology, Chinese Academy of Sciences, Beijing, China; University of Kentucky, UNITED STATES

## Abstract

Outbreaks of locust plagues result from the long-term accumulation of high-density egg production. The migratory locust, *Locusta migratoria*, displays dramatic differences in the egg-laid number with dependence on population density, while solitarious locusts lay more eggs compared to gregarious ones. However, the regulatory mechanism for the egg-laid number difference is unclear. Herein, we confirm that oosorption plays a crucial role in the regulation of egg number through the comparison of physiological and molecular biological profiles in gregarious and solitarious locusts. We find that gregarious oocytes display a 15% higher oosorption ratio than solitarious ones. Activinβ (*Actβ*) is the most highly upregulated gene in the gregarious terminal oocyte (GTO) compared to solitarious terminal oocyte (STO). Meanwhile, *Actβ* increases sharply from the normal oocyte (N) to resorption body 1 (RB1) stage during oosorption. The knockdown of *Actβ* significantly reduces the oosorption ratio by 13% in gregarious locusts, resulting in an increase in the egg-laid number. Based on bioinformatic prediction and experimental verification, microRNA-34 with three isoforms can target *Actβ*. The microRNAs display higher expression levels in STO than those in GTO and contrasting expression patterns of *Actβ* from the N to RB1 transition. Overexpression of each miR-34 isoform leads to decreased *Actβ* levels and significantly reduces the oosorption ratio in gregarious locusts. In contrast, inhibition of the miR-34 isoforms results in increased *Actβ* levels and eventually elevates the oosorption ratio of solitarious locusts. Our study reports an undescribed mechanism of oosorption through miRNA targeting of a TGFβ ligand and provides new insights into the mechanism of density-dependent reproductive adaption in insects.

## Author summary

The continuous accumulation of high-density eggs laid by flying swarms of adults results in huge populations of flightless juveniles, which contributes to the outbreaks of locust plagues. An interesting phenomenon is that locusts have the phenotypic plasticity of reproduction. The gregarious locusts lay fewer big eggs than do solitarious phase locusts. In contrast, the solitarious phase locusts lay more small eggs compared to the gregarious locusts. We find the egg-laid number is not only regulated by the phase status of parents but also controlled by oosorption, a type of oocyte death. Further studies confirmed the phase-related ratio of oocyte death in the mother is regulated by a microRNA, which posttranscriptionally influences the expression level of a TGFβ ligand. This maternal effect on progeny size is especially critical for gregarious locusts to control the population size and maintain population fitness, and for solitarious locusts to enhance chance for gregarization and further enlargement of population size. This is the first study to reveal the molecular mechanism underlying the regulation of a microRNA-gene circuit for locust oocyte death to determine the offspring number. These findings can provide some important clues to develop potential drugs to prevent vast locust reproduction from a plague upsurge.

## Introduction

Locusts can cause serious economic losses in agriculture upon population explosion. The accumulation of high-density egg numbers is one of the main causes of locust plague outbreaks. The migratory locust *Locusta migratoria* displays remarkable reproductive plasticity in the trade-off between egg size and clutch size (number of eggs per egg pod) between gregarious and solitarious ones [1, 2]. The high-density gregarious locusts lay larger but a little bit fewer eggs, while low-density solitarious locusts lay smaller but relatively more eggs [1, 3–5]. The egg-laid number is limited by the number of ovarioles and ratio of oocyte resorption, while solitarious females and their progeny hatchlings have higher numbers of ovarioles than their gregarious counterparts of *L*. *m*. *migratorioides* and *Schistocerca gregaria* [6, 7]. Meanwhile, oosorption (oocytes resorption), a process by which oocytes stop yolk deposition and are resorbed instead of being laid in response to behavioral, ecological or physiological factors, decreased the number of matured oocytes for egg production [8]. However, whether the egg-laid number difference is associated with ovariole number and/or oosorption in the migratory locust is still unclear.

Follicle degeneration, known as oosorption or follicular atresia, commonly occurs in insects and mammals. In mammals, follicular atresia is mainly regulated by the transforming growth factor β (TGFβ) superfamily members-initiated cascades. Several ligands, such as activin, bone morphogenetic proteins and Nodal have key roles in multiple aspects of follicle development [9]. These ligands are a large class of evolutionarily conserved polypeptides, exerting a wide range of biological effects by regulating cell growth, differentiation and cell fate determination [10]. In insects, follicle degeneration is mediated by several cellular processes, such as autophagy, apoptosis and necrosis [11, 12]. However, the upstream regulatory factors of oosorption are still unknown.

Our previous studies confirm that the phase transition of the migratory locust is regulated at the transcriptional and posttranscriptional levels. Several phase-core genes specifically regulate phenotypic plasticity in the behavior, body color, immunity and reproduction of the locusts [1, 13–18]. Expression differences of some coding genes and miRNAs confirm the linkages of phase traits in gregarious and solitarious locusts [19–22]. The gregarious behavior of the locust is regulated by dopamine and the genes in its synthesis pathway including *henna*, *pale* and *dop1* [14, 15]. Further study found that low miR-133 levels in gregarious locust resulted in high expression of *henna* and *pale* to synthesize more dopamine, maintaining locust aggregation [18]. As a dopamine receptor, *Dop1* activation enhanced adenylyl cyclase 2 expression by inhibiting miR-9a maturation to initiate olfactory attraction among locust individuals [13]. In particular, individual aggregation can promote the accumulation of more miR-276 in gregarious ovaries, synchronizing egg-hatching by upregulating *brm* by trans-generational inheritance [23]. Thus, the interactions between genes and miRNAs play critical roles in reproductive plasticity. However, whether gene and miRNA interactions control the egg-laid number difference between gregarious and solitarious locusts remains unclear.

In the present study, we investigate the relationships among the number of ovarioles, oosorption of ovary and egg-laid number in gregarious and solitarious migratory locusts, *Locusta migratoria migratoria*, and examine the differential regulation mechanism of egg production in response to population density changes. Our results demonstrated that different oosorption ratios in gregarious and solitarious locusts contributed to the differentiation of egg-laid number through miR-34 to target *Actβ*, regulating oosorption. Therefore, our study elucidated a molecular mechanism of egg number differences in response to population density in migratory locusts.

## Results

### The oosorption ratio is different in gregarious and solitarious locusts

**Fig 1:**
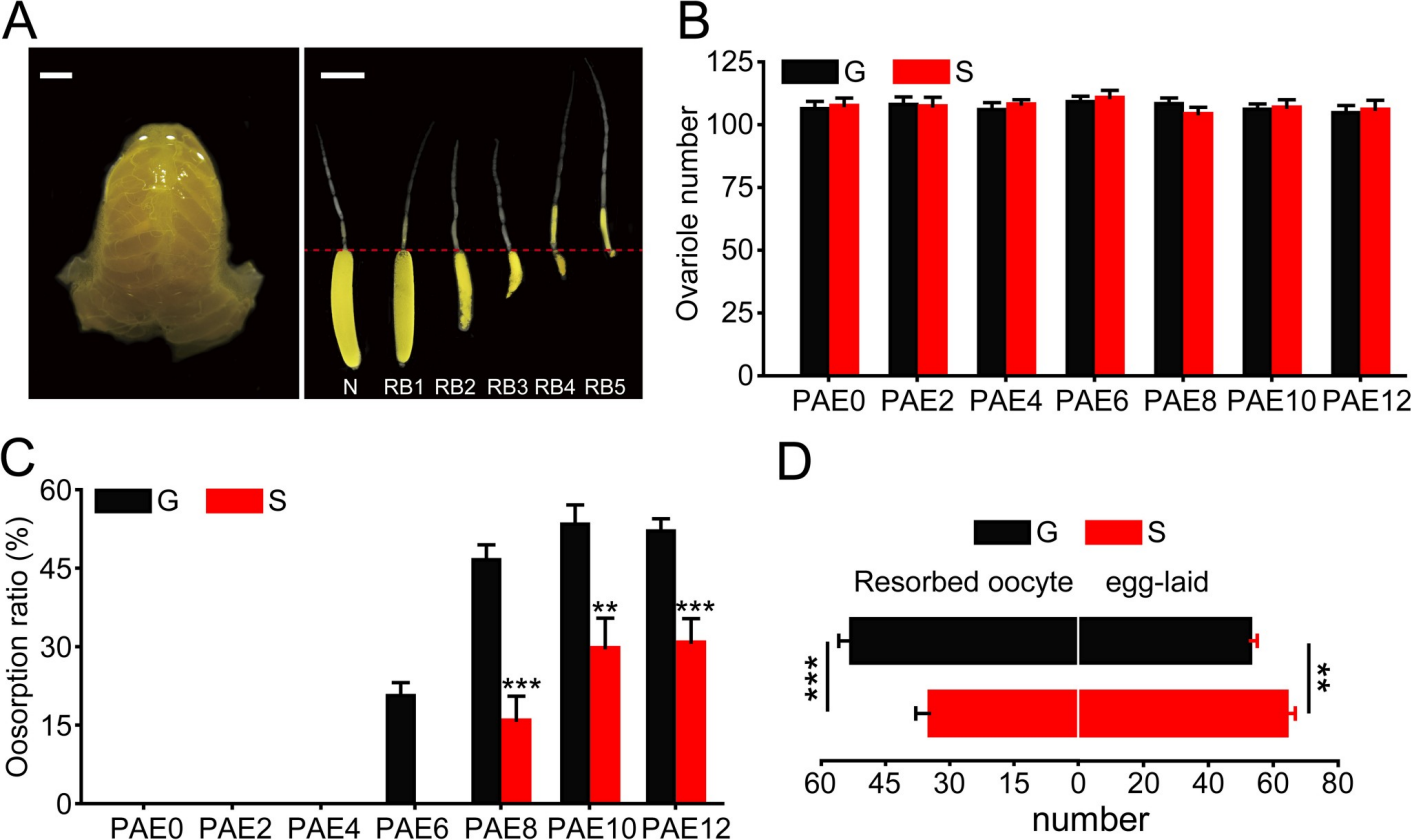
Oosorption differences between gregarious and solitarious locusts. (A) Normal and resorbed terminal oocytes at different stages. N, normal terminal oocyte; RB, resorption body. Scale bar, 2 mm. (B) Ovariole number of gregarious or solitarious adults at PAE0, 2, 4, 6, 8, 10 and 12. n = 15. (C) Oosorption ratio of gregarious or solitarious adults at PAE0, 2, 4, 6, 8, 10 and 12. n = 10–18. (D) The number of resorbed terminal oocytes and eggs in the first egg pod in the gregarious or solitarious adults. n = 15–16. **P < 0.01, ***P < 0.001.

Because the difference in the egg number in *L*. *m*. *migratorioides* and *S*. *gregaria* is mainly attributed to different ovariole numbers [6, 7], we investigate the differences in ovariole number and oosorption ratio between gregarious and solitarious *L*. *m*. *migratori*a under the same rearing conditions except for density. We found that the ovariole number showed no significant differences at 0, 2, 4, 6, 8, 10 and 12 days post adult eclosion (PAE0, 2, 4, 6, 8, 10, 12) between gregarious and solitarious locusts (Fig 1A and 1B). Then, we discriminated the oosorption into five stages in the locusts (Fig 1A) based on specific criteria [24, 25]. Compared to solitarious oocytes, gregarious ones showed significantly higher oosorption ratios of 21%, 31%, 24% and 21% from PAE6, 8, 10 and 12, respectively, during oocyte maturation (Fig 1C). Corresponding to the higher oosorption ratio, gregarious locusts laid 17% fewer eggs than solitarious locusts (Fig 1D). Thus, egg number variation is mainly affected by the oosorption ratio but not ovariole number in the migratory locust.

### *Actβ* promotes oosorption

**Fig 2:**
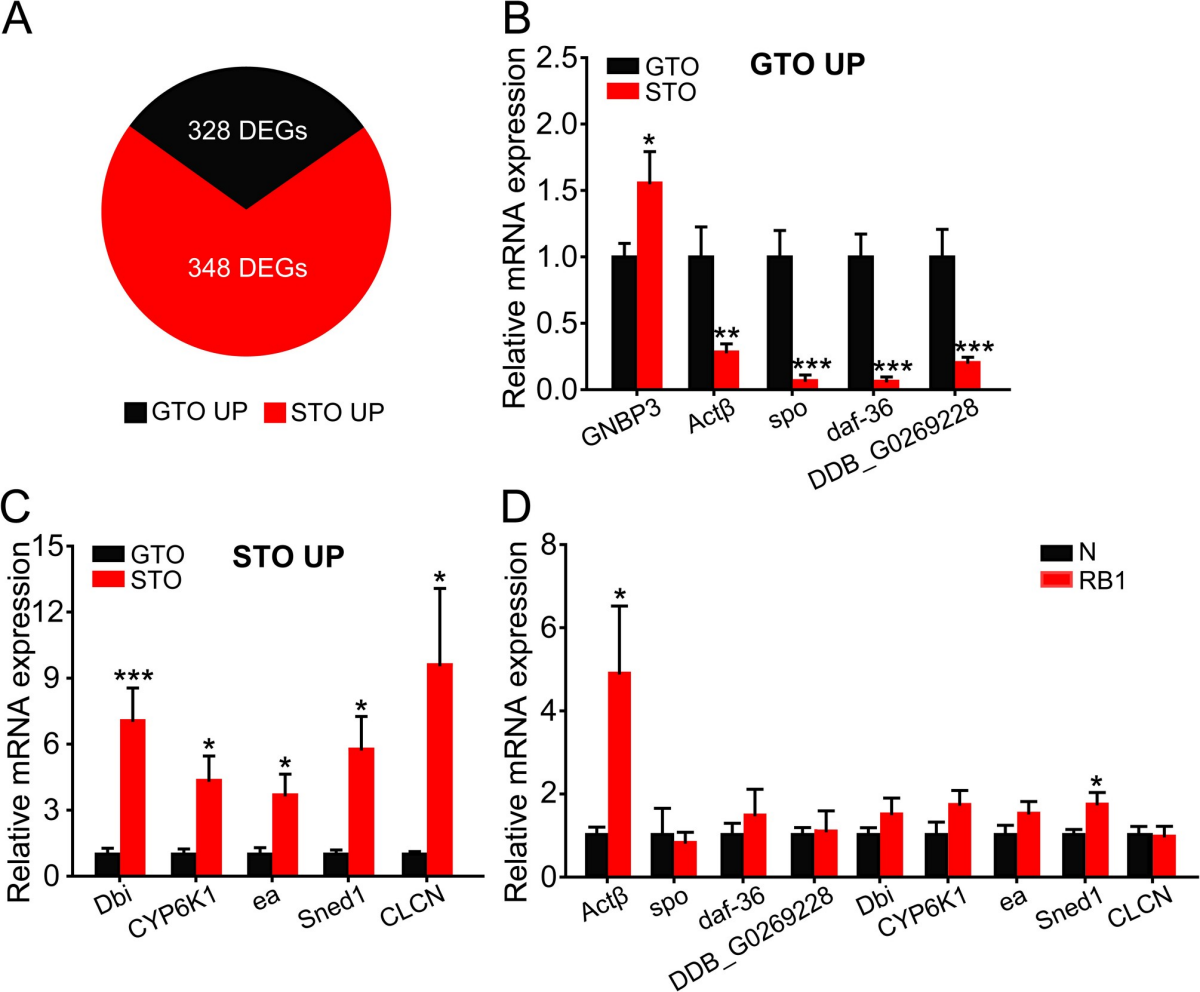
Transcriptomic analyses of gregarious (G) and solitarious (S) terminal oocytes (TO). (A) The number of differentially expressed genes between GTO and STO. (B and C) qRT-PCR verification of the top 5 genes upregulated in GTO (B) or STO (C). n = 7–12. (D) The expression levels of nine verified genes at N and RB1 stages. n = 4–7. *P < 0.05, **P < 0.01, ***P < 0.001.

To investigate which genes are involved in oosorption differences, we performed high-throughput RNA-seq to compare the gene expression profiles of gregarious terminal oocytes (GTO) and solitarious terminal oocytes (STO). In total, 676 genes were differentially expressed, and of these, 328 and 348 genes were upregulated in GTO and STO, respectively (S1 Dataset, Fig 2A). The top 5 genes that were upregulated in GTO or STO were selected as candidates and validated by qRT-PCR (Table 1). Gram-negative bacteria binding protein 3 (*GNBP3*), Activinβ (*Actβ*), Cytochrome P450 307a1 (*spo*), Cholesterol desaturase daf-36 (*daf-36*) and PI-PLC X domain-containing protein DDB_G0269228 (*DDB_G0269228*) were significantly upregulated in GTO (Fig 2B), and Acyl-CoA-binding protein homolog (*Dbi*), Cytochrome P450 6k1 (*CYP6K1*), Serine protease easter (*ea*), Sushi, nidogen and EGF-like domain-containing protein 1 (*Sned1*) and Epithelial chloride channel protein (*CLCN*) were significantly upregulated in STO (Fig 2C). Then, we compared the expression patterns of these 9 differentially expressed genes between normal (N) and resorption body 1 (RB1) oocytes. *Actβ* and *Sned1* showed significant increases of 4.9- and 1.7-fold in RB1 compared to N, respectively (Fig 2D). In combination with the respective expression patterns of *Actβ* and *Sned1* between GTO and STO, *Actβ* is considered a key candidate for regulation of the oosorption ratio in the locust.

**Table 1:** Top 5 genes upregulated in GTO or STO.

	Gene ID	GTO FPKM	STO FPKM	Log_2_FC (GTO/STO)	P value	Gene Name
GTO UP	LOCMI00582	33.87	1.07	4.98	4.58E-02	Gram-negative bacteria binding protein 3
	LOCMI16458	5.26	0.24	4.45	2.52E-02	Activinβ
	LOCMI16206	751.97	40.24	4.22	3.70E-02	Cytochrome P450 307a1
	LOCMI00790	230.22	13.49	4.09	7.58E-03	Cholesterol desaturase daf-36
	LOCMI05839	47.92	3.01	3.99	3.25E-02	PI-PLC X domain-containing protein DDB_G0269228
STO UP	LOCMI11326	7.09	163.51	-4.53	1.52E-02	Acyl-CoA-binding protein homolog
	LOCMI16169	0.47	5.25	-3.48	3.73E-02	Cytochrome P450 6k1
	LOCMI17544	0.92	9.86	-3.42	1.51E-04	Serine protease easter
	LOCMI14056	0.50	4.68	-3.22	4.30E-03	Sushi, nidogen and EGF-like domain-containing protein 1
	LOCMI10397	4.19	35.99	-3.10	1.03E-02	Epithelial chloride channel protein

**Fig 3:**
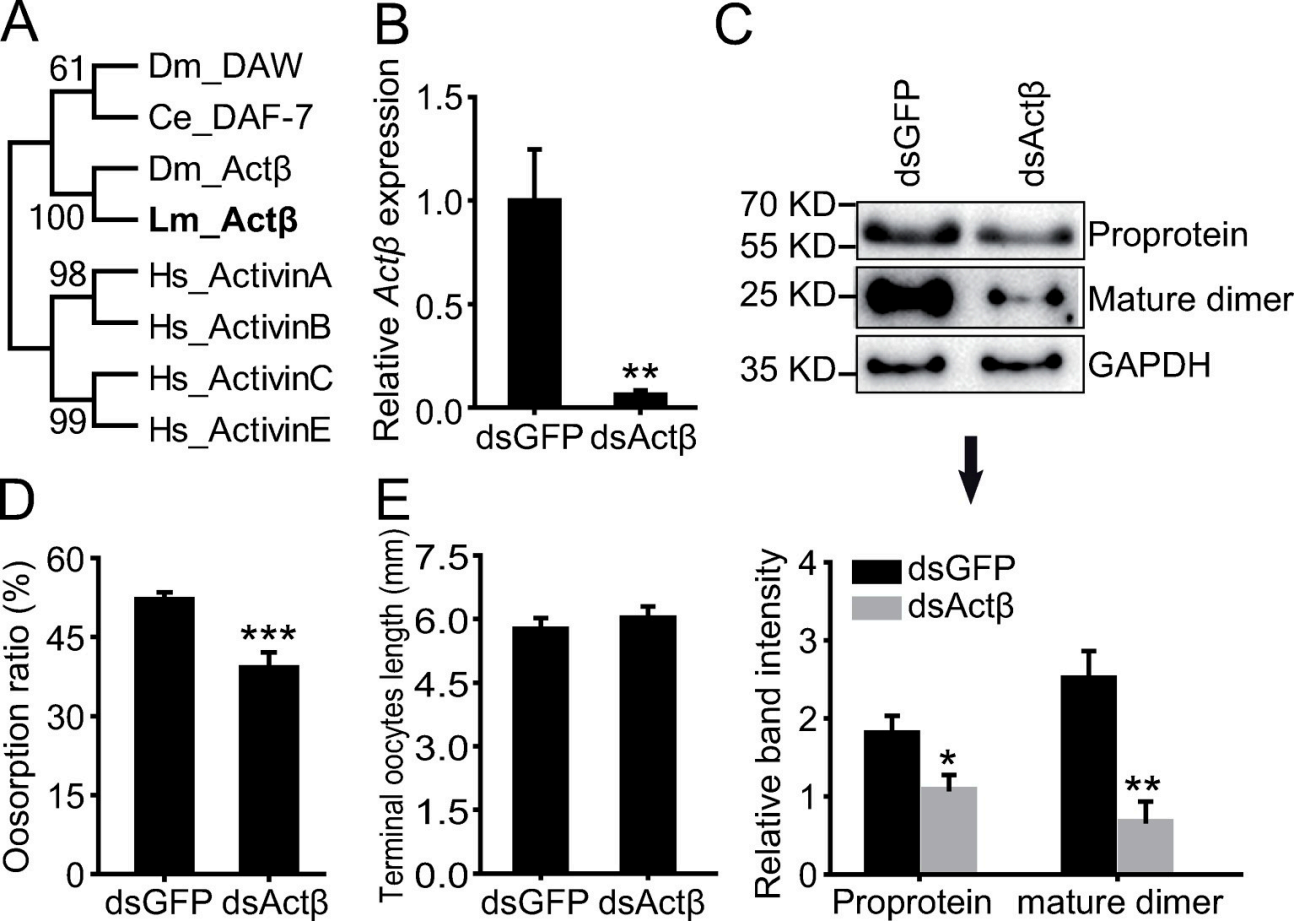
Effects of *Actβ* knockdown on oosorption. (A) Neighbor joining tree construction of TGFβ ligand subfamily in four species. Consensus unrooted trees were generated with 1000 bootstrap trials using the neighbor-joining method. Lm, *Locusta migratoria*; Ce, *Caenorhabditis elegans*; Dm, *Drosophila melanogaster*; Hs, *Homo sapiens*. (B) Gene expression level of *Actβ* in the terminal oocytes after dsGFP or dsActβ injection. n = 9–10. (C) Protein expression level of *Actβ* in the terminal oocytes after dsGFP or dsActβ injection. n = 4. (D) Oosorption ratio after dsGFP or dsActβ injection. n = 18. (E) Length of terminal oocytes after dsGFP or dsActβ injection. n = 8–9. *P < 0.05, **P < 0.01, ***P < 0.001.

*Actβ* belongs to the activin subfamily of TGFβ (Fig 3A). To confirm the function of *Actβ* in oosorption, we injected dsActβ in the gregarious female adults. The expression levels of *Actβ* mRNA, proprotein and mature dimer were significantly reduced to 6%, 60% and 27% of the dsGFP-injected controls in the terminal oocytes, respectively (Fig 3B and 3C). Consistently, the oosorption ratio was significantly decreased by 13% after dsActβ injection when compared to dsGFP-injected controls (Fig 3D). However, the terminal oocyte length was not significantly different between the two groups (Fig 3E), implying no significant effect on the egg size of the locusts.

### *Actβ* is a target of miR-34

**Fig 4:**
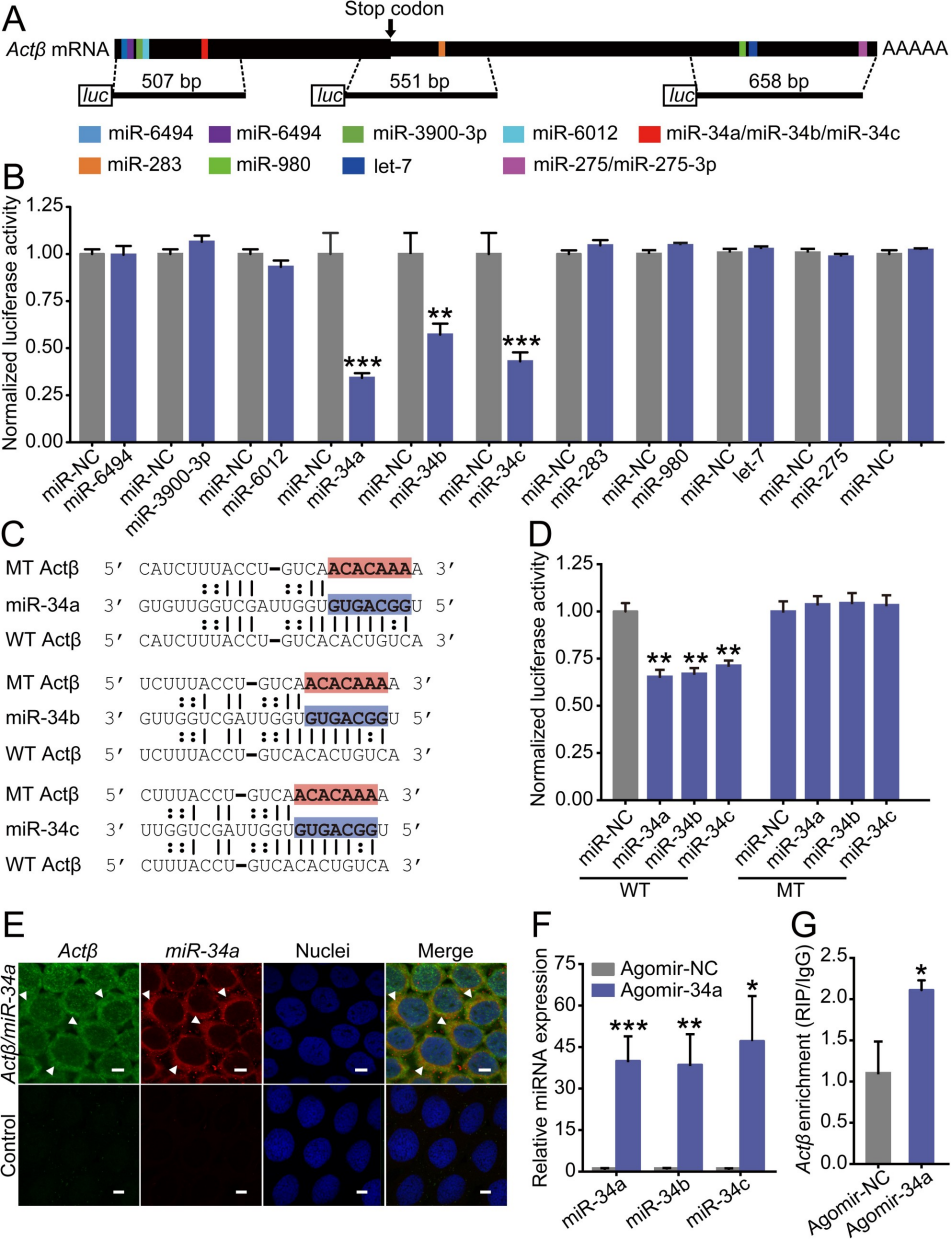
Identification of miRNAs targeting *Actβ*. (A) Diagram of miRNA target sites on the *Actβ* mRNA sequence. Three DNA fragments with target sites were inserted into psiCHECK-2 vectors for dual-luciferase reporter assays. (B) Dual-luciferase reporter assays using S2 cells cotransfected with miRNA mimics and recombinant psiCHECK2 vectors containing the predicted binding sites of respective miRNAs. miR-NC, negative control. n = 5–6. (C) The binding sites (in red) of Actβ were mutated into bases that were noncomplementary to the miR-34 seed region (in blue). WT, predicted binding sites; MT, mutated binding sites. (D) Dual-luciferase reporter assays after site mutation. n = 6. (E) Colocalization of miR-34a and *Actβ* by FISH in the follicle cells of terminal oocytes. Green, *Actβ*; red, miR-34a. Arrows indicate *Actβ*/miR-34a colocalized areas. Scale bar: 10 μm. *P < 0.05, **P < 0.01, ***P < 0.001. (F) The relative miRNA abundance of miR-34b or miR-34c in the terminal oocytes after agomir-NC or agomir-34a injection. n = 8–9. (G) The relative abundance of precipitated *Actβ* mRNA by Ago1-RIP in the terminal oocytes after agomir-NC or agomir-34a injection. n = 4.

Since posttranscriptional regulation plays variant roles in locust phase trait determination [13, 18, 23], we speculate that the expression difference of *Actβ* between gregarious and solitarious adult locusts is regulated by miRNA. Previously, 689 lineage-specific miRNAs and 144 evolutionarily conserved miRNAs were identified in the genome of *L*. *migratoria* [26]. Conserved miRNAs show sequence and target conservation among the entire metazoan kingdom [27]. Thus, based on these 144 conserved miRNAs, we predicted the potential miRNAs to target *Actβ* in the CDS and 3' UTR sequences by using the algorithms miRanda [28] and RNAhybrid [29]. In total, we obtained 11 predicted miRNAs, of which miR-6494, miR-3900-3p, miR-6012, miR-34a, miR-34b and miR-34c can bind to the CDS sequence, and miR-283, miR-980, let-7, miR-275 and miR-275-3p can bind to the 3’UTR sequence (Fig 4A, S1 Fig). In luciferase reporter assays in *Drosophila* S2 cells, we confirmed only miR-34a, miR-34b and miR-34c significantly decreased the luciferase activity by 66%, 43%, and 57% compared to the negative control miR-NC (Fig 4B). After mutating the complementary sequences of *Actβ* to the “seed region” of three miR-34 isoforms, the respective luciferase activities significantly recovered compared to S2 cells cotransfected with the wild-type psiCHECK-2 conducts and showed no difference compared with S2 cells cotransfected with miR-NC (Fig 4C and 4D). To investigate whether miR-34 colocalized with *Actβ in vivo*, we performed double fluorescence in situ hybridization (FISH) in the normal terminal oocytes. Due to the high similarity of these three miR-34 isoforms, we selected miR-34a as a representative, which has the highest expression level among them. The results showed that miR-34a and *Actβ* colocalized in the cytoplasm of follicle cells of the terminal oocytes (Fig 4E). Then, an RNA immunoprecipitation (RIP) assay from a monoclonal antibody against the Ago1 protein was used to examine the interaction of miR-34 with *Actβ in vivo*. Agomir-34a injection significantly increased the abundance of miR-34a, miR-34b and miR-34c in the terminal oocytes, resulting in significant enrichment of *Actβ* mRNA in the Ago1-immunoprecipitated RNAs (Fig 4F and 4G). Therefore, miR-34 can target and interact with *Actβ in vitro* and *in vivo*.

### miR-34 suppresses oosorption by downregulating *Actβ*

**Fig 5:**
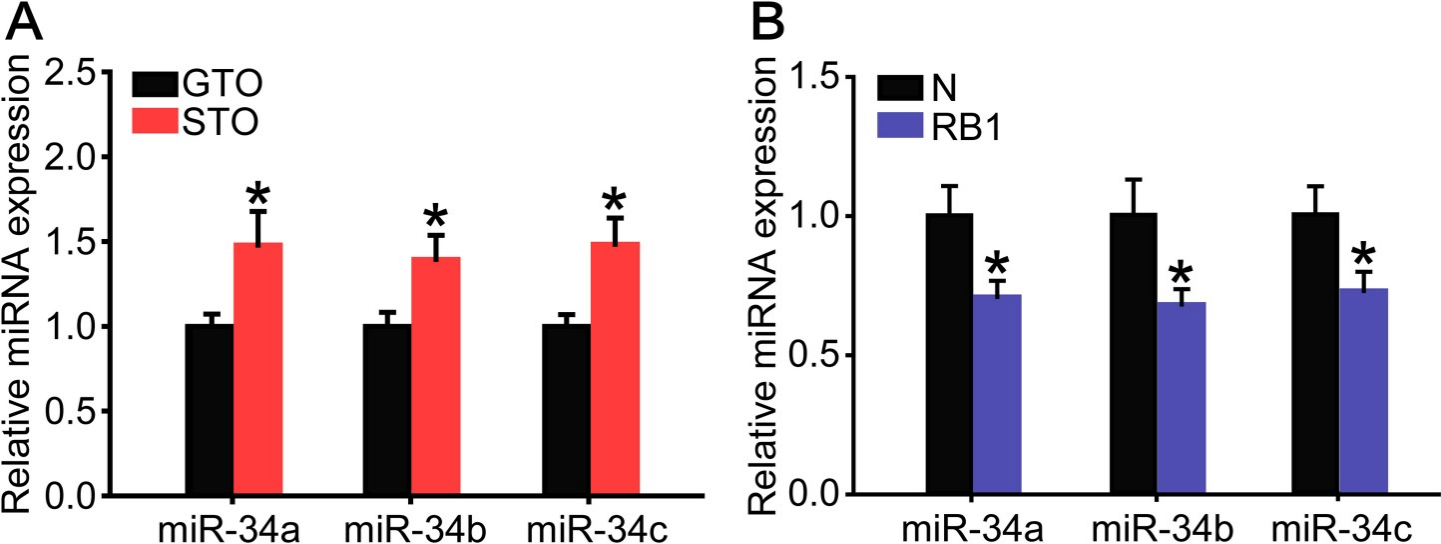
**The expression levels of three miR-34 isoforms in the GTO and STO (A) or in the N and RB1 (B).** (A) n = 12–15. (B) n = 6–8. *P < 0.05.

The expression levels of miR-34a, miR-34b and miR-34c were 1.5-, 1.4- and 1.5-fold higher in the solitarious locusts than in the gregarious ones in the N oocytes (Fig 5A). In contrast, the expression levels of miR-34a, miR-34b and miR-34c significantly decreased by 29%, 32%, and 28% in the RB1 when compared to N oocytes, respectively (Fig 5B). Thus, miR-34 showed a contrary expression pattern with the *Actβ* gene from N to RB1.

**Fig 6:**
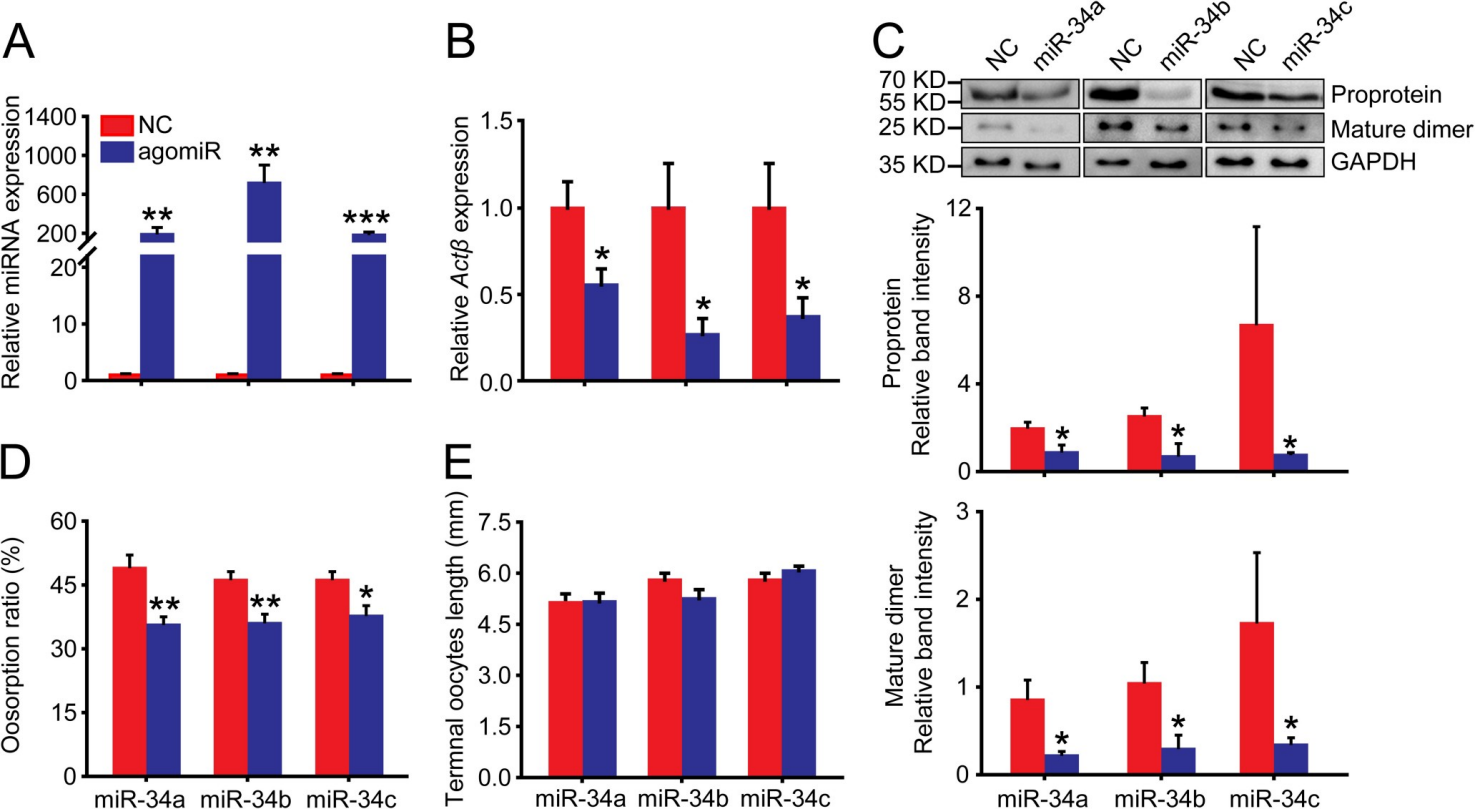
Effects of agomir-34 injection on oosorption in the gregarious locusts. (A) The relative miRNA abundance of miR-34a, miR-34b or miR-34c in the terminal oocytes after injection with respective agomirs. n = 7–8. (B) Gene expression level of *Actβ* in the terminal oocytes after injection with agomir of miR-34a, miR-34b or miR-34c. n = 5–8. (C) Protein expression level of *Actβ* in the terminal oocytes after injection with agomir of miR-34a, miR-34b or miR-34c. n = 4. (D) Oosorption ratios after injection with agomir of miR-34a, miR-34b or miR-34c. n = 10–13. (E) Length of terminal oocytes after injection with agomir of miR-34a, miR-34b or miR-34c. n = 8–13. *P < 0.05, **P < 0.01, ***P < 0.001.

We injected miR-34a, miR-34b and miR-34c agomirs in the gregarious females to enhance their expression levels to 189-, 716- and 182-fold, respectively, compared to the agomir-NC control in the terminal oocytes (Fig 6A). After injection with the three agomirs, the *Actβ* mRNA levels significantly decreased to 55%, 31% and 43% of the agomir-NC control, respectively (Fig 6B). The *Actβ* proprotein levels significantly decreased to 44%, 27% and 11% of the agomir-NC control, respectively (Fig 6C), and the *Actβ* mature dimer levels significantly decreased to 25%, 28% and 20% of the agomir-NC control, respectively (Fig 6C). Consistently, the oosorption ratios significantly decreased by 13%, 10% and 8% compared to the agomir-NC control, respectively (Fig 6D). However, the length of terminal oocytes was not significantly different from the agomir-NC control (Fig 6E).

**Fig 7:**
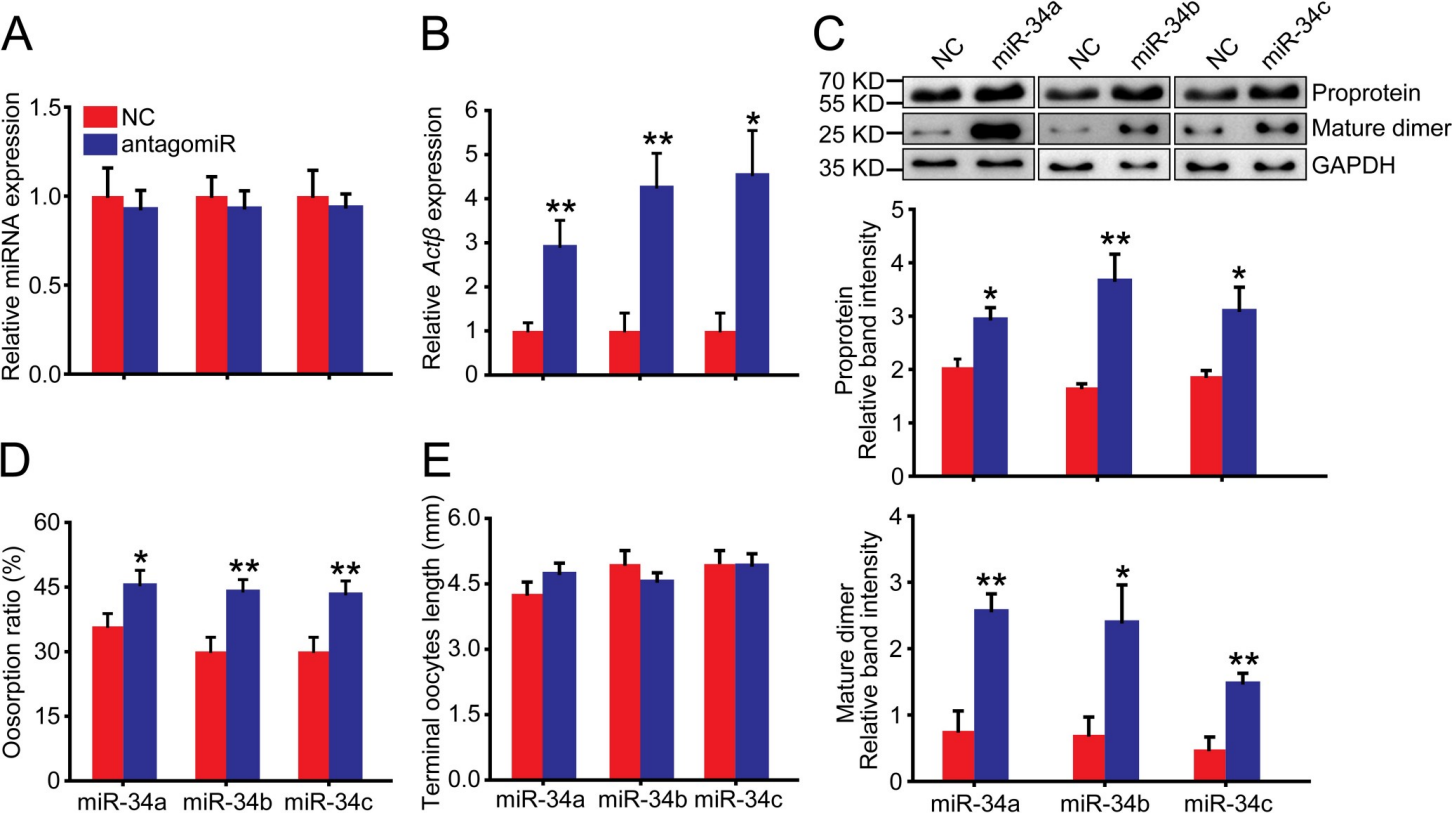
Effects of antagomir-34 injection on oosorption in the solitarious locusts. (A) The relative miRNA abundance of miR-34a, miR-34b or miR-34c in the terminal oocytes after injection with respective antagomirs. n = 7–8. (B) Gene expression level of *Actβ* in the terminal oocytes after injection with antagomir of miR-34a, miR-34b or miR-34c. n = 8–17. (C) Protein expression level of *Actβ* in the terminal oocytes after injection with antagomir of miR-34a, miR-34b or miR-34c. n = 4. (D) Oosorption ratios after injection with antagomir of miR-34a, miR-34b or miR-34c. n = 17–25. (E) Length of terminal oocytes after injection with antagomir of miR-34a, miR-34b or miR-34c. n = 9–17. *P < 0.05, **P < 0.01, ***P < 0.001.

In the solitarious female adults, we injected miR-34a, miR-34b and miR-34c antagomirs. The abundance of miR-34a, miR-34b and miR-34c showed no significant changes compared to antagomir-NC control in the normal terminal oocytes (Fig 7A). However, after injection with the three antagomirs, the *Actβ* mRNA levels significantly increased to 2.9-, 4.3- and 4.5-fold of the antagomir-NC control, respectively (Fig 7B). The *Actβ* proprotein levels significantly increased to 1.4-, 2.2- and 1.7-fold of the antagomir-NC control, respectively (Fig 7C), and the *Actβ* mature dimer levels significantly increased to 3.4-, 3.4- and 3.1-fold of the antagomir-NC control, respectively (Fig 7C). Correspondingly, the oosorption ratios significantly increased by 10%, 14% and 13% compared to the antagomir-NC control, respectively (Fig 7D). Similarly, the length of terminal oocytes was not significantly different from that of the antagomir-NC control (Fig 7E). Taken together, the oosorption ratios in the locust determine that the egg-laid number is regulated by the *Actβ* expression difference, which is a consequence of posttranscriptional modification due to miR-34 involvement.

## Discussion

Previous studies have shown that the difference in the number of ovarioles is phase-related and contributes to the larger clutch size in solitarious females than in gregarious ones in *L*. *m*. *migratorioides* and *S*. *gregaria* [6, 7]. However, our results found that the number of ovarioles was not different between gregarious and solitarious adult females of the migratory locust, *L*. *m*. *migratoria*. Nevertheless, fewer oocytes were resorbed in the solitarious adults, which resulted in a higher number of eggs laid by solitarious adults than gregarious adults. Our previous study confirmed that *Syx1A* indirectly determined progeny egg size and clutch size by regulating yolk protein in the hemolymph and ovary [1]. In this study, we showed that density-dependent regulation of the miR-34-*Actβ* circuit had a direct effect on oosorption but not the oocyte size in the terminal oocytes. Based on the adaptive investment hypothesis, progeny egg size is determined by maternal generation in response to environmental cues [30]. The adjustment of egg size inclusively leads to egg number changes for reallocation of limited nutritional resources under high population density [1]. The molecular signal miR-34-*Actβ*, possibly as an alternative for condition-dependent trade-off in reproductive plasticity, regulates the respective high or low oosorption ratio in gregarious or solitarious locusts for environmental adaptation.

The *L*. *m*. *migratoria* adopted the oosorption adjustment strategy but not both the oosorption and ovariole number adjustment strategy as in the *L*. *m*. *migratorioides* and *S*. *gregaria* to control progeny number [6, 7]. The causes of the differential adjustment strategies might reside in three aspects. First, some phase traits displayed species-specific characteristics, such as how aggregation behavior is controlled by dopamine in *L*. *migratoria* [14, 15] but by serotonin in *S*. *gregaria* [31]. In the grasshopper *Melanoplus sanguinipe*, parental crowding did not affect the ovariole number [32]. In the grasshopper *Romalea microptera*, the ovariole number was influenced by genetic variation and nutrition during development, but not maternal environment [33]. Second, differential internal physiological states might affect the final strategy. In *S*. *gregaria*, studies revealed contrasting oocyte development rates and JH titers between gregarious and solitarious adults [34, 35]. Third, habitat may contribute to the different strategies. The African migratory locust, *L*. *m*. *migratorioides*, and desert locust, *S*. *gregaria*, live in tropical and subtropical zones; however, *L*. *m*. *migratoria* is mainly distributed in the temperate zone. The study of 17 populations of coho salmon (*Oncorhynchus kisutch*) in North America showed a significant latitudinal increase in their egg number, but this increase was accompanied by significant latitudinal decreases both in egg size and total biomass of eggs produced [36]. The tropical butterfly *Bicyclus anynana* exhibited temperature-mediated plasticity in egg size and number, laying higher numbers of smaller eggs at the higher temperature but fewer and larger eggs at the lower temperature [37]. We inferred that the differential strategies adopted by variant species or subspecies might be the results of regulation by different genes or pathways.

Our study showed that *Actβ* acted as a regulator of oosorption in the migratory locust. Although the TGFβ ligand-initiated pathways are conserved from *Caenorhabditis elegans* (*C*. *elegans*) to *Homo sapiens*, they function in variant aspects of reproduction [9, 38]. The regulatory roles of TGFβ ligands in folliculogenesis are rather complex among species because abundant family members are involved in [9]. In *C*. *elegans*, reduced TGFβ Sma/Mab signaling from *dbl-1* positively regulates germline/oocyte quality maintenance through *sma-2* [39]. In *Drosophila*, *gbb* and *dpp* are essential for GSC maintenance in the ovary [40] and *dpp* also functions in the formation of eggshell structure [41]. In mammals, activin A fosters granulosa cell proliferation, preantral follicle growth and antral formation, as well as increase in the survival of preantral follicles by decreasing the proportion of atretic follicles [42, 43]. Another ligand, Nodal, was found to promote granulosa cell apoptosis and follicular atresia [44]. In migratory locusts, *Actβ*, which is a homolog of mammalian activin, fly Actβ and worm DAF-7 (Fig 3A), has special function in regulating oocyte death during oosorption.

Furthermore, we found that *Actβ* was regulated by the three miR-34 isoforms in the locusts. miR-34 belongs to a markedly conserved miRNA family and has three mammalian homologs and a single ortholog in invertebrate species with identical seed sequence [45]. In *Drosophila*, miR-34 displays three isoforms (miR-34a, miR-34b and miR-34c) that have a uniform 5′ terminus but differ at the 3′ end resulting from nibbler-mediated trimming, where nibbler trims the 24 nt mR-34a for the generation of shorter isoforms of 22 nt miR-34b and 21 nt miR-34c with preferred lengths for Ago1 binding [46]. The sequences of the locust miR-34 isoforms are identical to that of *Drosophila*. With agomir-34a treatment in the locust, the miRNA abundance of miR-34a as well as the shorter miR-34b and miR-34c isoforms all increased significantly (Fig 4F). Additionally, treatment with agomir-34b in the locust resulted in significant increases in miR-34c abundance (S2 Fig). Our results suggested the mechanism of miR-34 isoforms production might be similar to that in *Drosophila*. The three isoforms of miR-34 jointly downregulated *Actβ* expression.

Because of the different physiological and behavioral characteristics, gregarious and solitarious locusts present obviously different allocation of resources as a life-history trait trade-off. Gregarious locusts allocate more resources in flying and migration rather than reproduction like in other migratory insects [47–49]. However, non-migratory solitarious locusts tend to invest more resources in reproduction through more egg-laying for enlargement of population size. Therefore, gregarious and solitarious locusts balance the resources allocation through differential oosorption ratios. Oosorption determines the clutch size between gregarious and solitarious locusts, which provides alternative strategy for environmental adaptation. In addition, vitellogenesis-driven oocyte maturation is energy-consuming for massive syntheses and transportation of vitellogenin and other proteins [50, 51], thus oosorption also provides alternatives for the reallocation of resources in variant insect species under varied conditions such as food stress [8, 52]. The elucidation of miRNA-mediated regulation on TGFβ signaling in locusts is particularly crucial for understanding the reproduction plasticity and reallocation of body energy of insects and potentially provides new targets for pest control.

## Materials and methods

### Insects

Gregarious and solitarious locusts were from the same colonies maintained at the Institute of Zoology, Chinese Academy of Sciences, China. Approximately 200–300 gregarious locusts were kept in a cage (25 cm×25 cm×25 cm), and solitarious locusts were cultured alone in small metal cages. Both colonies were reared under a 14:10 light/dark photo regime at 30±2°C. The locusts were fed with fresh wheat seedlings and bran every day. The gregarious male locusts were removed from the cage immediately post adult eclosion, and the females were continually kept at high density.

### Transcriptomic sequencing and data processing

Fifteen terminal oocytes from three individuals of gregarious or solitarious locusts were pooled together respectively as one biological replicate and three biological replicates were collected for transcriptomic sequencing. Total RNA was extracted using TRIzol reagent (Invitrogen) according to the manufacturer’s instructions. Approximately 3 μg of total RNA was used to establish a paired-end RNA-seq library for transcriptome sequencing on an Illumina HiSeq 2000 platform (Tiangen). After the low-quality reads had been trimmed and adapters had been removed, the clean RNA-seq reads were mapped to the reference genome using Tophat2 (version 2.0.13) with default parameters [26, 53, 54]. The unique mapped reads were used to calculate the number of reads that mapped to an aviary gene model using HTSeq [55]. The influence of differences in RNA output size between samples was reduced by the trimmed mean of M-values (TMM) method [56]. Gene expression level was measured as fragments per kilobase million (FPKM). Student’s t test was used for the significance test. Genes with significance levels of P < 0.05 and fold change ≥ 2 were considered differentially expressed. The RNA-seq data was deposited in the Sequence Read Archive database (accession no. PRJNA659804).

### qRT-PCR verification

Each sample of total RNA was isolated from five normally developed terminal oocytes or resorption body 1 oocytes per female adult respectively as one biological replicate using TRIzol reagent (Invitrogen). For mRNA quantification, Oligo (dT)-primed cDNA was reverse transcribed from total RNA using Moloney murine leukemia virus (M-MLV) reverse transcriptase (Promega). qRT-PCR was carried out using a LightCycler 480 instrument (Roche) and Talent qPCR PreMix (SYBR Green) (Tiangen) according to the manufacturer’s instructions. For miRNA quantification, the miRcute Plus miRNA First-Strand cDNA Synthesis Kit (Tiangen) was used to synthesize the cDNA. The cDNA of miRNA was subjected to qRT-PCR using the miRcute Plus miRNA qPCR Detection Kit (SYBR Green) (Tiangen) according to the manufacturer’s instructions on a LightCycler 480 instrument (Roche). The PCR data were analyzed by the 2^-△△Ct^ method of relative quantification with the internal control *rp49* and U6 for mRNA and miRNA, respectively. All the primers for qRT-PCR are listed in S1 Table.

### RNA interference (RNAi)

Double stranded RNAs of *Actβ* and green fluorescent protein (GFP) were synthesized *in vitro* using the T7 RiboMAX Express RNAi System (Promega). For RNAi in locusts, a total of 8 μg (1 μg/μL) of *Actβ* or *GFP* dsRNA dissolved in a mixture of acetone and H_2_O (2:1 ratio) was intra-abdominally injected into each female adult within 12 h after eclosion and boosted on day 5 [57]. Upon maturation, the oosorption ratio was calculated by the total number of RB1 to RB5 oocytes divided by the total number of terminal oocytes in an individual locust, and five terminal oocytes per female adult were sampled as one biological replicate and stored in liquid nitrogen for qRT-PCR analysis. The primers for dsRNA synthesis are listed in S1 Table.

### Luciferase reporter assays

The 507 bp, 551 bp and 658 bp sequences of CDS and 3′UTR surrounding the predicted target sites of miR-6494, miR-3900-3p, miR-6012, miR-34 miR-283 miR-980, let-7, miR-275 and miR-275-3p in *Actβ* were separately cloned into the psiCHECK-2 vector (Promega) using the Xho I and Not I sites. Site mutation in the *Actβ* DNA sequence complementary to the miR-34 seed region was performed by using the KOD-Plus-Mutagenesis Kit (TOYOBO). The mimic of *C*. *elegans* miRNA, cel-miR-67-3p (5′ to 3′: UCACAACCUCCUAGAAAGAGUAGA), was used as the negative control (miR-NC) [23]. *Drosophila* S2 cells with complete Schneider’s *Drosophila* medium were cotransfected with the luciferase reporter vector (WT or MT) and miRNA mimics or miR-NC using lipofectamine 3000 (Invitrogen). Luciferase activity was determined using the Dual-Luciferase Reporter Assay System and a GloMax 96 Microplate Luminometer (Turner Biosystems Instrument). Normalized luciferase activity was calculated by dividing Renilla luciferase activity by firefly luciferase activity and normalizing to the mean of the control miR-NC group.

### RNA immunoprecipitation (RIP) assay

The RIP assay was performed using a Magna RIP Quad kit (Millipore) with slight modifications. Briefly, approximately 40 terminal oocytes were collected as one biological duplication and homogenized in ice-cold RIP lysis buffer. The homogenates were stored at -80°C overnight. After centrifugation for 15 min at 14,000 ×g, the supernatant was incubated at 4°C overnight with magnetic beads preincubated with a monoclonal antibody against locust Ago1 [18] or normal mouse IgG. The precipitated RNA was quantified by qRT-PCR.

### Fluorescence In Situ Hybridization (FISH)

The RNA probe for *Actβ* was synthesized by a T7/SP6 RNA Transcription Kit (Roche) and was subsequently fragmented to approximately 250 bp by carbonate buffer. The primers used for probe synthesis of *Actβ* are in S1 Table. Ovarioles were separated from ovaries in locust saline and fixed in 4% (wt/vol) paraformaldehyde overnight. After digestion with proteinase K (20 μg/mL; Tiangen) at 37°C for 15 min, these ovarioles were hybridized with miR-34a probe (2 pmol/mL) and *Actβ* probe (5 ng/μL) at 37°C overnight. Then, the ovarioles were successively washed in 2× SSC, 1× SSC, and 0.2× SSC at 37°C. Anti-DIG alkaline phosphatase-conjugated antibody (1:100) and anti-biotin antibody (1:100) were used for probe detection. Then, the fluorescent signal of digoxigenin (DIG) or biotin was obtained by HNPP/Fast Red (HNPP Fluorescent Detection Set, Roche) or Fluorescein-Tyramide (TSA Fluorescein System, Perkin Elmer). Images were captured on an LSM 710 confocal fluorescence microscope (Carl Zeiss) at a magnification of 63×.

### miRNA agomir and antagomir treatments

Agomir-34a, agomir-34b and agomir-34c are chemically modified double-strand stable miRNA mimics. Antagomir-34a, antagomir-34b and antagomir-34c are chemically modified single-strand stable miRNA inhibitors whose sequences are reverse complementary to respective miR-34 isoforms. Agomir and antagomir of cel-miR-67-3p (agomir-NC and antagomir-NC, respectively) were used as negative controls. The agomirs and antagomirs were synthesized by GenePharma, Inc. (Suzhou). Adult females within 12 h after eclosion were intra-abdominally injected with 2.5 μg of miRNA antagomir or agomir mixed with RNA Transfection Reagent (Engreen) and boosted at PAE5. Upon maturation, the oosorption ratio was calculated in each individual locust, and five terminal oocytes per female adult were sampled as one biological replicate and stored in liquid nitrogen for qRT-PCR analysis.

### Western blotting

The polyclonal antibody for *Actβ* was raised against a synthetic peptide (CTPPLEEYRKMDSLP) mapping at the C-terminus of *Actβ* and produced from rabbits. Total proteins were extracted by TRIzol reagent (Invitrogen). The proteins were subjected to polyacrylamide gel (10%) electrophoresis and then transferred to polyvinylidene difluoride (PVDF) membranes (Millipore). Blocking was performed in 5% (wt/vol) skimmed milk at room temperature (RT) for 1 h. The membranes were incubated with primary antibody (anti-Actβ, 1 μg/mL; anti-GAPDH, 1:5000) in 5% (wt/vol) skimmed milk at 4°C overnight. Secondary antibody (1:5,000) (CWBIO) was incubated at RT for 1 h. The immunological blot was detected using SuperSignal™ West Femto Maximum Sensitivity Substrate (Thermo Scientific). Protein band intensities were quantified using Image-Pro Plus 6.0 software (Media Cybernetics Inc.).

### Statistical analysis

Statistical analysis was performed by Student’s t-test and the nonparametric Mann–Whitney *U* test using IBM SPSS Statistics v.19 software (SPSS Inc.). Differences were considered significant at *P*<0.05. Values are reported as the mean±SE.

## Supporting information

S1 Table Primers used for qRT-PCR, dsRNA synthesis and probe synthesis. (DOCX)

S1 Fig Prediction of miRNAs that bind to *Actβ*. In red, the seed region of miRNA.
(TIF)

S2 Fig The relative miR-34c abundance in the terminal oocytes after injection with agomir-34b. n = 7–8. **P < 0.01.
(TIF)

S1 Dataset Differentially expressed genes between gregarious and solitarious terminal oocytes. (XLSX)
