# Supplementary material for: Phase-related differences in egg production of the migratory locust regulated by differential oosorption through microRNA-34 targeting activinβ
Source: PLoS Genet. 2021 Jan 6;17(1):e1009174. doi: 10.1371/journal.pgen.1009174 (PMC7787450; doi:10.1371/journal.pgen.1009174)
Supplement: S1 Table — (DOCX) [file pgen.1009174.s001.docx]

S1 Table. Primers used for qRT-PCR, dsRNA and FISH probe synthesis

| **Primer name** | **Sequence (5**′ **to 3**′**)** |
| --- | --- |
| **qRT-PCR** |  |
| GNBP3F | ACGACGCCGTTACGACAG |
| GNBP3R | TCACCTCCAAGTTCAAAATGTTT |
| Actβ-F | TGTAAACCTGGTAGGCTGTCC |
| Actβ-R | TTACTGTCGTGGTGATTGTGG |
| spoF | GGCTTGTGTCGCTGTTCG |
| spoR | TAGGTCTCGCCGCTGTTG |
| daf-36F | GTTATTCATCGCATATTCTGTCCT |
| daf-36R | AGTGGCTTATCTTCGTATTTCTTG |
| DDB_G0269228F | GAGGAGGTGTGGTGGCTGA |
| DDB_G0269228R | ACGGGGAACTCGTGGAAG |
| DbiF | TCGAGCTGTACGCGCTCT |
| DbiR | CTCCTTGGCTTCATCCTTTG |
| CYP6K1F | CTACTGGCAGACCAAGGGC |
| CYP6K1R | GAAGCAAAGGACGACGAAAT |
| eaF | GTGGCGGCGTGCTCATCA |
| eaR | CGCTCCAGGTCCGTGTTGT |
| Sned1F | GAGACAAAAACTCAGATGAATACCA |
| Sned1R | GCAGGACACACACAGCGAAA |
| CLCNF | GAAATCCTTCCCTCCTCGAT |
| CLCNR | CCCACTTACTGGCCCGTC |
| rp49-F | CGTAAACCGAAGGGAATTGA |
| rp49-R | GAAGAAACTGCATGGGCAAT |
| U6-F | ACACTCCAGCTGGGTCAAAATCGTGAAGCG |
| miR-34a | TGGCAGTGTGGTTAGCTGGTTGTG |
| miR-34b | TGGCAGTGTGGTTAGCTGGTTGA |
| miR-34c | TGGCAGTGTGGTTAGCTGGTTA |
| **dsRNA** |  |
| dsActβ-F | AGGCTGTCCATCTTTCTATACTCC |
| dsActβ-R | TTAAACGGGCAGACGCTCC |
| dsGFP-F | CACAAGTTCAGCGTGTCCG |
| dsGFP-R | GTTCACCTTGATGCCGTTC |
| **FISH probe** |  |
| T7-Actβ-F | GGATCCTAATACGACTCACTATAGGAGAGATGGAGGGCGAGGG |
| Actβ-R | GCTGAAAAGATAAGAATGTGAGTGT |
| Actβ-F | AGAGATGGAGGGCGAGGG |
| T7- Actβ-R | GGATCCTAATACGACTCACTATAGGGCTGAAAAGATAAGAATGTGAGTGT |
