# Supplementary figures and images for: Phase-related differences in egg production of the migratory locust regulated by differential oosorption through microRNA-34 targeting activinβ
Source: PLoS Genet. 2021 Jan 6;17(1):e1009174. doi: 10.1371/journal.pgen.1009174 (PMC7787450; doi:10.1371/journal.pgen.1009174)

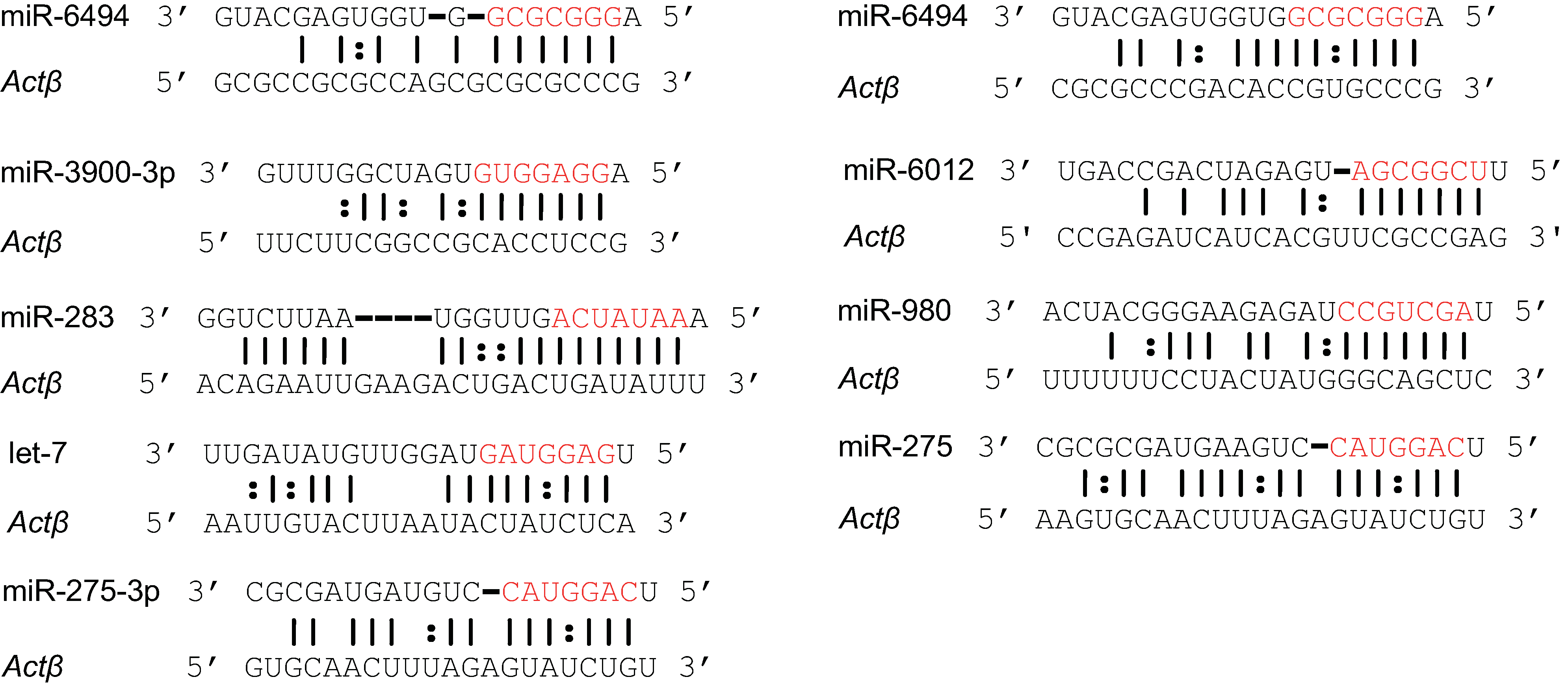

Supplement: S1 Fig — In red, the seed region of miRNA. (TIF) [file pgen.1009174.s002.tif]

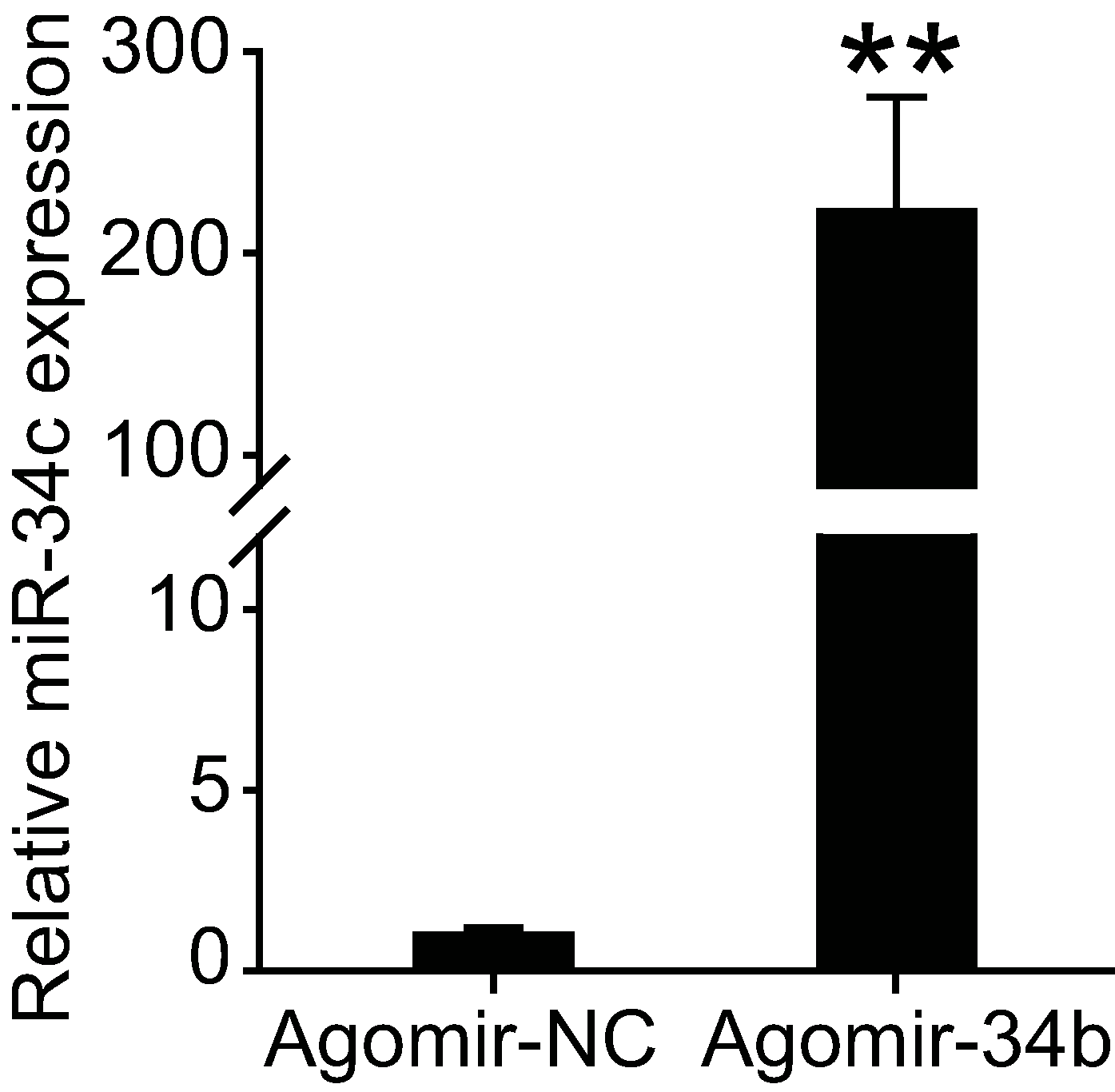

Supplement: S2 Fig — n = 7–8. **P < 0.01. (TIF) [file pgen.1009174.s003.tif]
